# Seasonal Variations in the Culturable Mycobiome of *Acropora loripes* along a Depth Gradient

**DOI:** 10.3390/microorganisms8081139

**Published:** 2020-07-28

**Authors:** Nofar Lifshitz, Lena Hazanov, Maoz Fine, Oded Yarden

**Affiliations:** 1 Department of Plant Pathology and Microbiology, The Robert H. Smith Faculty of Agriculture, Food and Environment, The Hebrew University of Jerusalem, Rehovot 76100, Israel; nofarlif@gmail.com; 2 The Interuniversity Institute for Marine Science, P.O.B. 469, Eilat 88103, Israel; lenka8@gmail.com (L.H.); Maoz.Fine@biu.ac.il (M.F.); 3 The Goodman Faculty of Life Sciences, Bar-Ilan University, Ramat Gan 52900, Israel

**Keywords:** mycobiome, marine fungi, coral holobiont

## Abstract

Coral associated fungi are widespread, highly diverse and are part and parcel of the coral holobiont. To study how environmental conditions prevailing near the coral-host may affect fungal diversity, the culturable (isolated on potato dextrose agar) mycobiome associated with *Acropora loripes* colonies was seasonally sampled along a depth gradient in the Gulf of Aqaba. Fragments were sampled from both apparently healthy coral colonies as well as those exhibiting observable lesions. Based on phylogenetic analysis of 197 fungal sequences, Ascomycota were the most prevalent (91.9%). The abundance of fungi increased with increasing water depth, where corals sampled at 25 m yielded up to 70% more fungal colony forming units (CFUs) than those isolated at 6 m. Fungal diversity at 25 m was also markedly higher, with over 2-fold more fungal families represented. Diversity was also higher in lesioned coral samples, when compared to apparently healthy colonies. In winter, concurrent with water column mixing and increased levels of available nutrients, at the shallow depths, *Saccharomytacea* and *Sporidiobolacea* were more prevalent, while in spring and fall *Trichocomacea* (overall, the most prevalent family isolated throughout this study) were the most abundant taxa isolated at these depths as well as at deeper sampling sites. Our results highlight the dynamic nature of the culturable coral mycobiome and its sensitivity to environmental conditions and coral health.

## 1. Introduction

The estimate of marine-derived fungal species has been put at over 10,000 [1] and documentation concerning the occurrence of fungal associations with marine life forms is accumulating [2,3,4]. Fungi are now considered part and parcel of the microbiome present in a variety of constituents of the coral reef [5,6]. A growing number of studies report on the presence and isolation of fungi from corals and sponges, using culture- and DNA sequencing-based techniques [7,8,9,10]. Some of these have been shown to have ecological significance in microbe-microbe and microbe-host interactions [11,12,13]. An in-depth metagenomic/transcriptomic analysis of fungi associated with the coral *Acropora hyacinthus* has demonstrated the presence of a diverse, metabolically active, fungal community and has also uncovered a phylogenetically diverse core assemblage of associated fungi [11]. Metagenomic analysis of bleached *A. millepora* revealed a 3- fold increase in fungal-like sequences, yet the role of fungi during this stress event is unclear [12]. In another scleractinian coral, *Porites compressa*, a change in the relative ratio of colonies comprising the coral microbiome was shown to occur following an acute stress event [13]. The authors suggested that this is indicative of a shift from a mutualistic and/or commensal community to one that may be more potentially pathogenic and/or opportunistic. Changes in the spatial distribution of *A. formosa*-associated fungi (at least 2 of them novel species [14]) have also been shown to occur under stress conditions [15].

Over the past decades, coral reefs of the world are experiencing an increase in the occurrence of coral syndromes and diseases [5,16]. Nonetheless, most of the primary pathogens remain unknown and only a few causative agents of the described diseases have been identified. Even then, in many instances, disease can be a combined outcome of pathogen virulence, environmental impacts and changes in host immune response [5]. The only fungal coral pathogen currently fulfilling Koch’s postulates is *Aspergillus sydowii,* which causes aspergillosis among sea fan corals (*Gorgonia ventalina* and *G. flabellum*) in the Caribbean [17,18], yet it has also been isolated (with no observable symptoms) from apparently healthy sea fans [19] as well as a marine sponge–*Spongia obscura* [20]. Aspergillosis outbreaks have occurred during the summer months when high seawater temperature have been suggested to enhance fungal growth rate, possibly assisting it to overcome the host’s defenses [21,22]. These findings suggest that changing environmental conditions might have a vast impact on the health of marine ecosystems.

Using a culture-based approach, we explored fungal diversity in a common Red Sea Acroporid coral, *Acropora loripes*, conducting a survey of the natural fungal community in coral colonies from different depths and during different time points throughout the year. The survey included fungal distribution in healthy coral specimens as well as those exhibiting observable lesions, which may be the result of predation and physical damage by divers as well as pathogens. Our findings demonstrate a seasonal variation in fungal communities associated with apparently-healthy coral versus those exhibiting lesions, along a depth gradient, highlighting the sensitivity of this association to environmental conditions. We suggest that the spatial and temporal surroundings can confer alterations in the coral mycobiome and, subsequently, may affect coral health.

## 2. Methods

### 2.1. Sampling Site and Coral Collection

**Figure 1:**
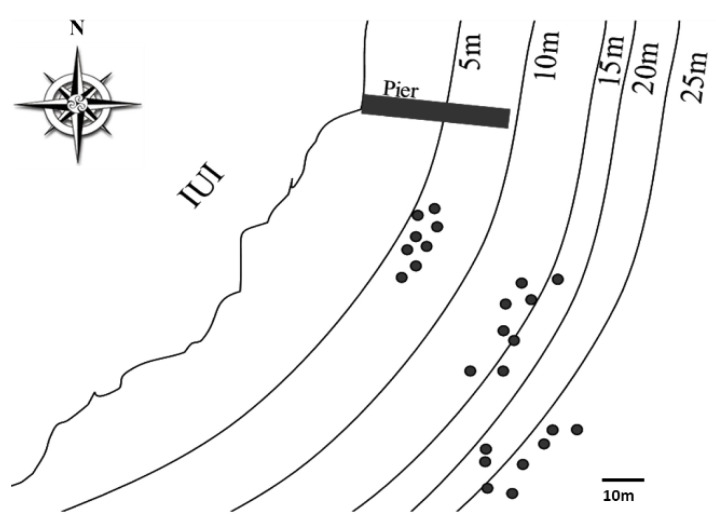
Map of the *Acropora loripes* sampling sites in front of the Interuniversity Institute for Marine Sciences (IUI), stating coral colony locations and depths.

Twenty-four colonies of *A. loripes* were selected and tagged at three depth ranges (5–6, 14–16 and 24–25 m) (Figure 1) at the reef adjacent to the Interuniversity Institute for Marine Sciences in Eilat (IUI), northern Red Sea (29.5°N, 34.9°E). At each site, four apparently-healthy colonies and four colonies that had observable lesions (5–15% of the colony tissue was damaged/diseased) were selected. Samples from the colonies were collected at three time points during different seasons, during September 2007, February 2008 and June 2008. Three, 3 cm, fragments were collected, by SCUBA, from each colony at these occasions. Samples from damaged/diseased colonies were collected from the necrotic area.

### 2.2. Sample Processing

Fragments were collected in sterile zip-lock bags and processed in a laminar flow hood to avoid contamination by air-borne fungal propagules. Each coral fragment (approx. 3 cm in length) was rinsed in about 100 mL of autoclaved and subsequently 0.2 µm-filtered (Millipore, Burlington, MA, USA) sea water (FSW) at ambient sea temperature (~22–25 °C). Washed fragments were gently crushed using a sterilized hammer and spread, in duplicates, on plates containing Potato Dextrose Agar (PDA, Difco, Franklin Lakes, NJ, USA) supplemented with 250 mg liter^−1^ chloramphenicol (PDACl). Control plates were left exposed, for 5 min, inside the hood during the processing of the samples to test for aerial contamination. In addition, several plates were sprayed, using an air-brush, with FSW to control for possible presence of contamination in the FSW used. All culture plates were incubated at 25 °C (ambient sea water temperature) until no additional fungal growth was evident and to a maximum period of seven days. Purification of fungal isolates was performed by repeated colony tip isolation on PDA.

Fungi were also isolated from water and sediment obtained from the sampling area. In order to concentrate fungal propagules from the sampled seawater, 200 mL of water were filtered using a sterile vacuum system (250 mL Filter System, Corning, Corning, NY, USA). The filter and sediment were plated on PDACl and incubated in a similar manner.

### 2.3. DNA Extraction and PCR Amplification

DNA extraction from pure fungal cultures was performed using a protocol modified from Zhou et al., [23]. Briefly, fungal mycelium was scraped from the culture plates and suspended in 2 mL tubes containing 200 µL de-ionized water (NANOpure, Barnstead Co., Newton, MA, USA) and an equal amount of 0.5 mm glass beads (acid washed, Sigma-Aldrich, St. Louis, MO, USA). The tube was agitated using a Mini Bead Beater (Biospec Products Inc., Bartlesville, OK, USA) for 100 s, followed by 10 min boiling to inactivate endogenous nucleases. The samples were cooled to room temperature and spun at 825× *g* for 2 min to pellet cell debris. The supernatant was used for PCR-based DNA amplification.

Fungal identification was based on partial 18S rRNA gene DNA sequence. The amplicons were obtained using primers KF1 (GTTGCAGTTAAAAAGCTCGTAGTYG) and KR1 (AATTGACGGAAGGGCACCA) which were designed using the Premier 5 software package. PCR was performed in a volume of 25 μL containing 1 μL DNA (0.2–2 ng/μL), 12.5 μL ReadyMix PCR Master Mix (Thermo Fisher Scientific, Dreieich, Germany), 1 μL forward primer (10 μM), 1 μL reverse primer (10 μM) and 9.5 μL of de-ionized water. The following program was carried out in a Primus 96 thermocycler (Peqlab, Fareham, UK): Initial denaturation of DNA at 94 °C for 4 min and then 35 cycles of three-step PCR amplifications consisting of denaturation at 94 °C for 1 min, primer annealing at 56 °C for 45 s, and extension at 72 °C for 45 s. The samples were subjected to an additional extension at 70 °C for 7 min at the end of the amplification cycle. PCR products were separated by agarose gel electrophoresis. Amplicons were purified (Wizard SV Gel and PCR Clean Up System, Promega, San Luis Obispo, CA, USA) and sequenced (Macrogen, Seoul, South Korea).

### 2.4. Phylogenetic and Diversity Analysis

The fungal amplicon sequences (GenBank accession numbers MT605585 - MT605801) were compared to the NCBI database using the BLAST algorithm (www.ncbi.nlm.nih.gov/BLAST). Multiple-sequence alignment was obtained with MEGA7 software utilizing the ClustalW program option which first conducts a pairwise alignment followed by a multiple-sequence alignment. For Phylogenetic tree construction, the evolutionary history was inferred by using the Maximum Likelihood method based on the Tamura-Nei model [24]. The tree with the highest log likelihood (−1374.33) is shown. The percentage of trees in which the associated taxa clustered together is shown next to the branches. Initial tree(s) for the heuristic search were obtained automatically by applying Neighbor-Join and BioNJ algorithms to a matrix of pairwise distances estimated using the Maximum Composite Likelihood (MCL) approach, and then selecting the topology with superior log likelihood value. The tree is drawn to scale, with branch lengths measured in the number of substitutions per site. The analysis involved 195 nucleotide sequences. Codon positions included were 1st + 2nd + 3rd + Noncoding. All positions containing gaps and missing data were eliminated. There were a total of 310 positions in the final dataset. Evolutionary analyses were conducted in MEGA7 [25]. 

The Shannon diversity index (H) was calculated to compare the structural diversity of the fungal communities in different depths and in apparently-healthy colonies along with colonies showing observable lesions.

## 3. Results

### 3.1. Fungal Diversity in A. loripes

**Figure 2:**
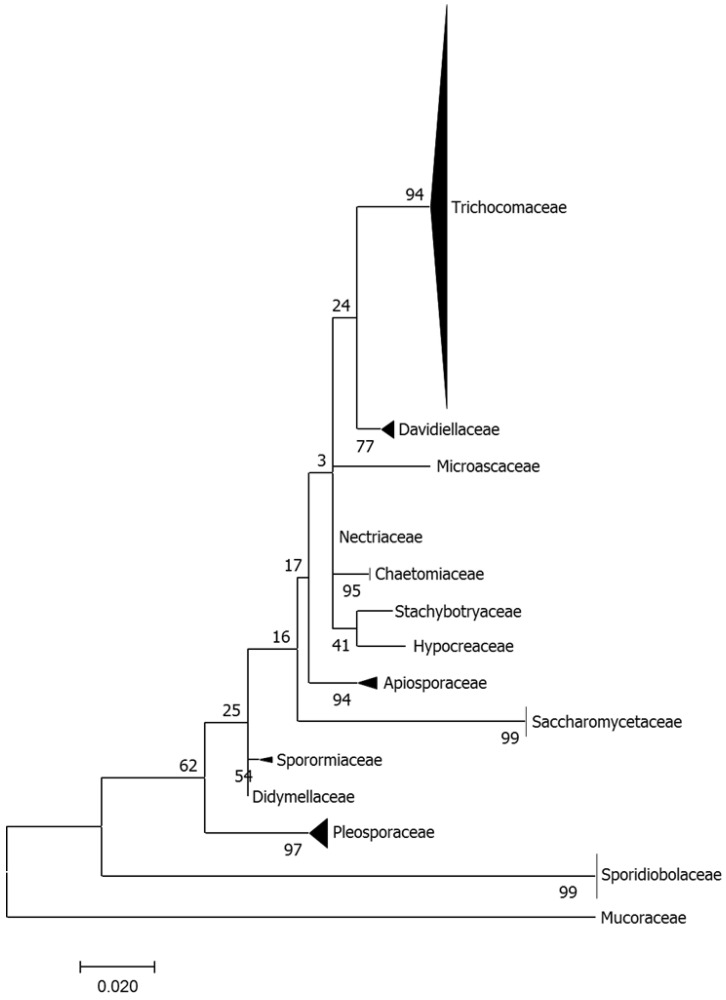
Phylogenetic tree describing the relative abundance of fungal family representatives isolated from *Acropora loripes*. Molecular Phylogenetic analysis was carried out by Maximum Likelihood method. The tree is drawn to scale, with branch lengths measured in the number of substitutions per site. Evolutionary analyses were conducted in MEGA7.

Overall, ninety-three fragments from 24 different coral colonies (*n* = 24) were collected, yielding about 200 fungal strains. The phylogenetic analysis based on the available 197 fungal sequences showed that members of the Ascomycota were the most prevalent (91.9%) while other phyla, the Basidiomycota and Zygomycota, accounted for only 7.6% and < 1% of the isolates, respectively. Based on partial 18S rDNA sequencing, the fungal strain collection was grouped to 14 families (Figure 2). *Trichocomaceae* was the most common and diverse family present, with 137 isolates representing genera of that family, predominantly *Aspergillus* and *Penicillium* spp. This was followed, albeit with a much lower overall representation, by genera belonging to the *Sporidiobolaceae* (mostly *Rhodotorula* spp.) and *Saccharomycetaceae* (*Candida* spp.) as well as additional genera (e.g., *Alternaria*, *Cladosporium*, *Chaetomium*, *Fusarium* spp.) representing other families.

### 3.2. Fungal Diversity in A. loripes along the Reef Slope

**Figure 3:**
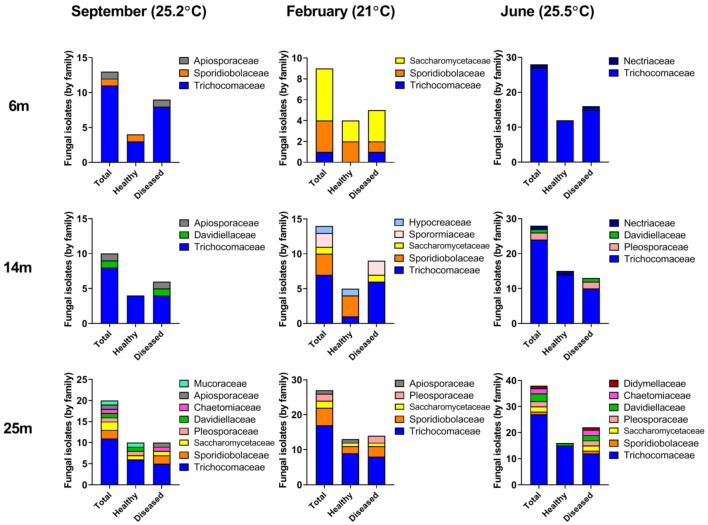
Prevalence of *Acropora loripes*-associated fungal families during the various seasons and along a depth gradient in the Red Sea. The total number of taxa, by family, as well as distribution between apparently-healthy and lesioned coral samples, is shown.

The abundance as well as the diversity of isolated fungal taxa varied in a manner that was dependent on the depth of the coral colonies. The most pronounced depth-dependent difference in the number of culturable fungi obtained was in samples from a depth of 25 m, which yielded an approximate 2-fold higher number of colonies than that observed in samples from the shallower sampling sites (Figure 3).The abundance of *Trichocomaceae* family members (as mentioned, the most abundant taxa isolated from *A. loripes*) increased from 39 isolates at 6 and 14 m to 55 isolates at 25 m. In addition to the increase in overall abundance, we also found an increase in diversity of the fungi isolated. The Shannon diversity index calculated for 25 m was 1.3125, vs. 1.0602 and 0.7826 calculated for 14 m and 6 m, respectively, indicating a higher diversity with depth. The number of families whose representatives were found increased from 5, at 6 m, to 9 at 14 m and 10 at 25 m. Furthermore, at the deepest sampling points, members belonging to 3 families (*Didymellaceae*, *Chaetomiacea* and *Mucoraceae*) not present in the shallower depths were identified. In summary, both the diversity and the abundance of fungi increased with increasing depth (Figure 3).

### 3.3. Seasonal Fluctuations in Fungal Diversity

**Figure 4:**
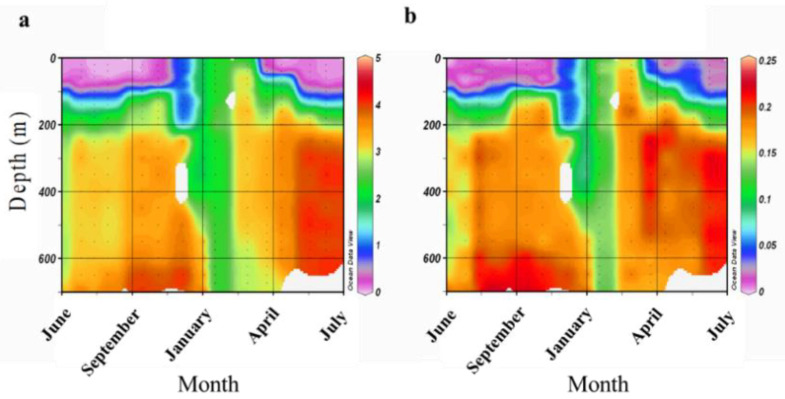
Nutrient variations measured in 2007–2008 at an open water site in the Gulf of Aqaba (northern Red Sea, N 29° 30.211’ E 34° 55.068’) throughout the survey. (**a**): Total Oxidized Nitrogen (TON) (micromole/l) and (**b**): Phosphates (PO_4_) (micromole/l). The data was obtained by the Israeli national monitoring program and processed by Ocean Data View (ODV) software.

Fungal abundance and diversity were also dependent on the time of year in which corals were sampled. We isolated an almost 2-fold higher number of strains when sampling in early summer (Figure 3). The number of strains belonging to the most prominent culturable component of the *A. loripes* mycobiome, the *Trichocomaceae*, increased their abundance between February and June by almost 3-fold. A similar trend was observed in the increased abundance of *Pleosporaceae* and *Davidiellaceae* between late summer and winter compared with early summer, even though the total number of representatives of these families was much lower (Figure 3). Yeast genera belonging to *Sporidiobolaceae* and *Saccharomycetaceae* were highly abundant in winter but rare during the summer samplings and their abundance decreased by ~75% between winter and summer. During summer, the yeasts were represented by the teleomorph genera *Candida* and *Rhodotorula*. It is conceivable that the differences in nutrient availability along the water column (Figure 4), which changes in accordance with depth of the mixed layer [26], may affect the spatial and temporal abundance of fungal species in the surveyed region.

### 3.4. Changes in Fungal Abundance and Diversity in Apparently-Healthy Corals vs. Corals Exhibiting Lesions

Diversity of the fungal community was evaluated in healthy colonies of *A. loripes* as well as in colonies which had macroscopically-visible lesions. The colonies showing observable lesions yielded a markedly higher number of fungi, 110, compared with 87 isolates cultured from the apparently-healthy corals. The prevalence of *Trichocomaceae* in healthy versus lesioned corals was 66 and 79 isolates, respectively. In general, the diversity of all the fungal families was higher in the coral samples presenting lesions and appeared to be more diverse at greater depths (Figure 3). This was also expressed by the lower Shannon diversity index calculated for the fungal community isolated from the apparently-healthy corals (1.035) as opposed to that of the colonies showing observable lesions (1.3999). In fact, members of 4 fungal families were found to occur only in corals displaying lesions. The overall increase in abundance and diversity of fungal taxa in lesioned corals may be indicative of opportunistic and/or saprophytic fungal activity, including the acquisition of additional taxa from the environment surrounding the lesioned coral. 

### 3.5. Presence of Fungi in Water and Sediment

The presence of fungi in water and sediment samples collected from the vicinity of the coral sampling areas was also monitored. Among the 20 fungal isolates that were cultured from these samples, 9 were identified as *Trichocomaceae*, which was the most common family found in the coral samples. Single culturable *Pleosporaceae* and *Nectriaceae* strains were also isolated. In addition, we also identified strains belonging to the *Onygenaceae*, *Botryosphaeriaceae* families, as well as 6 strains of the *Lulworthiaceae*, whose members are common marine ascomycetes [27]. No members of these families were found in the coral samples.

## 4. Discussion

The ecological significance of the coral mycobiome is far from understood, even though various roles of fungi in the marine environment have been proposed [28,29]. The present study is the first case where *A. loripes* has been examined for the presence of fungi. Even though this coral has not yet been a subject of mycobiome studies, the presence of a variety of taxa associated with this coral was not surprising, as fungi have been found in association with a broad spectrum of Cnidarians, Porifera and other sessile marine animals [2]. 

The associated fungal community recovered in this study increased in diversity and abundance with increasing depth. In addition, the abundance of different fungal families was higher in corals exhibiting lesions, when compared with apparently-healthy colony samples. 

Even though we were able to culture and identify a significant number of *A. loripes* mycobiome constituents, we believe that the range of fungal taxa harbored by this coral species is far from being fully described. We have also proposed this to be the case after isolating 85 taxa from a marine sponge [30]. Media used for isolation of marine fungi can vary immensely and can affect both the quantitative as well as qualitative efficacy of fungal strain isolation [20,30,31,32,33]. This is also likely to be the case with the medium used here, PDA. It is very likely that additional fungi, which are dependent on different nutritional as well as environmental conditions/requirements, are associated with this coral and it is also likely that we have not been successful in isolating rarer taxa. Furthermore, the possibility of obligate marine taxa (marine sensu strictu; [34]) present in *A. loripes* should not be ruled out. In addition, the nature of association of fungi with the host can vary. Some may be transient (e.g., short-term, superficial adherence or even ingestion) and some may reside in the tissue and/or proliferate into the skeletal cavity [2,15]. In this case, we assume that most, if not all of the repeatedly-isolated taxa are actual members of the mycobiome. Their physical location and distribution within the holobiont have yet to be determined.

While marine-derived fungi have been isolated from a variety of depths, comparative analyses of fungal diversity and abundance along a depth gradient is still scarce, especially with regard to association with corals. Recently, Morales et al., [35] conducted a thorough, global, metagenomic-based analysis of fungal diversity by depth and temperature and determined that depth was a structuring factor of community composition in marine fungi. We observed a similar trend in this local and sea depth-limited (standard SCUBA-accessible) coral-associated fungal analysis. Coral samples from the deeper part of the slope (25 m) exhibited the highest number of isolates throughout the survey. This was expressed by an increase in the abundance of *Trichocomaceae* as well as a higher diversity of several other, less common, taxa (Figure 3). These findings are in agreement with the high abundance of endolithic fungi found in carbonate substrates at similar depths habitats (30 m) in a previous study conducted in the Gulf of Eilat [36]. The main parameter that varies with depth is the light intensity and spectrum that governs the distribution and biomass of phototrophic populations [37]. Light intensity and quality may change the productivity of the coral and it’s photosymbiotic dinoflagellates [38], their photoacclimation through depth and seasons and in turn, the nutrients available to members of the holobiont.

When examining the seasonal changes in fungal family diversity, it is apparent that a marked change in the abundance of major constituents of the coral mycobiome were altered in the winter. Specifically, a reduction in the presence of *Trichocomaceae* was accompanied by an increase in predominantly yeast taxa of the *Saccharomytacea* and *Sporidibolacea* families. This change is highly correlated with a change in the distribution of available nitrogen and phosphorus along the depth gradient (Figure 4). The stratified layers of the water column undergo a seasonal mixing in the winter, resulting in the disruption of the nutrient gradient and an increase in the available nutrients at shallow depths. The Gulf of Aqaba water column stratification is highly sensitive to temperature decrease in surface water and mixing in winter may exceed 700 m (Figure 4) resulting in a substantial increase in nutrients supply to shallower layers [39] Hence, it is possible that the combination of environmental conditions (e.g., temperature and nutrient availability in the water column) may influence the diversity of the coral mycobiome. In a genome comparison analysis of Saccharomycotina and Pezizomycotina, Arvas et al., [40] found that genomes of the latter contain a higher abundance of metabolism clusters, among them, those encoding proteins involved in degradation of external nutrient sources such as polysaccharides which can be found in algae. Thus, the higher prevalence of *Saccharomytacea* and *Sporidibolacea* we observed in the winter may be a result of their ability to prosper only when limiting levels of nitrogen and phosphorus are more available (as occurs in that season). Consequently, once nutrients are scarcer again, an increase in the more metabolically-adaptable *Trichocomacea* (a family within the Pezizomycotina) is evident. Wegley et al., [41] analyzed the microbiome of *Porites astreoides* and reported on a wide array of fungal genes involved in carbon and nitrogen metabolism. They suggested that coral-associated fungi could be involved in converting nitrate and nitrite to ammonia, which would enable fixed nitrogen to cycle within the coral holobiont, even though in some cases nitrate has been shown to be assimilated directly by symbionts [42,43]. These processes could well be influenced by the fluctuations in availability of oxidized nitrogen in the water column. Hence, in addition to the various abiotic effects of the environment on the diversity of the fungi identified, it is also possible that seasonal physiological changes in the coral host affect the abundance and diversity of its mycobiome.

Corals presenting lesions exhibited a higher representation of the various families in comparison with apparently-healthy colonies. Similar observations were reported in bleached corals [12]. The higher number (approx. 20%) of fungal colonies isolated from stressed corals may be due to the possibility that these are more predisposed to fungal proliferation. The increase in fungal growth detected in lesioned corals agrees with a previous study on *Acropora spp.* from the Great Barrier Reef [15]. We also propose that under sub-optimal immune states, fungal propagule acquisition from the water column or adjacent reef dwellers may be more likely to occur. In fact, we have observed the presence of at least 4 taxa which were unique to the lesioned coral samples. One source of these may be rarer holobiont constituents which were now detected due to proliferation in the lesioned-coral. Another possibility is that these taxa are present in the environment (as has been the case here with members of at least 3 families present in the coral) and are more readily acquired by the stressed coral. Taken together, it may well be that, as in other animals, reduced host competence favors a shift from commensal to opportunistic lifestyles of resident fungi as well as species not usually associated with the host. We suggest that members of the *Trichocomaceae* constitute a major part of the core mycobiome of *A. loripes* (Figure 2). This is supported by the fact that presence of *Trichocomaceae* is barely influenced by the physiological state of the colony in apparently-healthy versus lesioned coral samples (Figure 3). While we have no evidence for any pathogenicity of the fungi isolated from *A. loripes*, at least some reports have suggested that coral-inhabiting fungi can be parasitic associates that attempt to attack corals’ polyps [44] and can even cause mass mortality [17]. Mera and Bourne [5], have described the challenge in assigning possible roles for coral microbiome members as actual pathogens, suggesting that coral health cannot always be attributed to a single causative agent. At the same time, just as mycobiome constituents have been proposed to contribute to nutrient acquisition/cycling [41], it is also possible that here too, some of them may have beneficial attributes for their host or other constituents of the host microbiome.

Roitman et al., [45] proposed that variations in coral microbiome assembly and functionality during changing environmental conditions may act as reliable bioindicators for coral-reef well-being. Regardless of their role, it is likely that identifying the components of the coral mycobiome and monitoring their fluctuations can be used, in the future, to assist in evaluating coral health and to aid in sustaining reef conservation. This may be especially relevant considering the growing evidence of the occurrence of coral disease and the alarming forecasts regarding global changes and their profound implications on the marine biota.
